# VKH-like uveitis during donafenib therapy for hepatocellular carcinoma: a case report and review of the literature

**DOI:** 10.3389/fphar.2024.1401017

**Published:** 2024-07-18

**Authors:** Rui Liu, Guina Liu, Fang Lu

**Affiliations:** Department of Ophthalmology, West China Hospital, Sichuan University, Chengdu, China

**Keywords:** VKH-like uveitis, donafenib-induced uveitis, hepatocellular carcinoma, targeted therapy, ocular side effects of cancer therapy

## Abstract

**Background:**

The incidence of uveitis has risen with the use of targeted therapies, particularly prevalent in the administration of immune checkpoint inhibitors and MAP-kinase pathway inhibitors. We report the first case of VKH-like uveitis linked to Donafenib employed for the primary hepatocellular carcinoma, highlighting the necessity of ophthalmological follow-up in patients undergoing treatment with Donafenib.

**Case presentation:**

A 55-year-old man developed VKH-like symptoms, including sporadic white patches, tinnitus, headache, and mild bilateral vision reduction, after 18 months of treatment with Donafenib and Sintilimab for hepatocellular carcinoma. Based on ophthalmological examinations that fundus fluorescein angiography images demonstrating multiple focal areas of pinpoint hyperfluorescence, along with pooling indicative of neurosensory detachment and disc leakage in both eyes, choroid thickening in swept-source optical coherence tomography, and “sunset-glow” fundus appearance, a tentative diagnosis of VKH-like uveitis was made. Initially, his best-corrected visual acuity (BCVA) was 20/200 in the right eye and 20/80 in the left eye. Upon discontinuing Donafenib and starting a 3-month course of oral glucocorticoids, his BCVA improved to 20/30 in the right eye and 20/40 in the left eye.

**Conclusion:**

Targeted drugs have been commonly used for cancer treatment in recent years, but challenges of ocular side effects emerged gradually. To optimize patient outcomes, regular ophthalmological follow-ups are essential for those undergoing treatment with targeted therapies like Donafenib.

## Background

Immune checkpoint inhibitors (ICIs) and MAP-kinase pathway inhibitors (MAPKis) are primarily responsible for the high occurrence of drug-induced uveitis (DIU) (Thibault et al., 2024). ICIs have been identified as causative agents for several ocular conditions, including dry eye, conjunctivitis, uveitis, scleritis and choroidal retinitis (Liu et al., 2020; Fortes et al., 2021). Ocular side effects associated with MAPKis primarily affect the retina and include chorioretinopathy and serous retinal detachment, leading the symptoms like blurred vision, halos around lights (Jeng-Miller et al., 2024; Thibault et al., 2024). Additionally, reports indicate that MAPKis cause ocular adverse effects more frequently than ICIs (Sada et al., 2023).

Donafenib, a novel small molecule multi-kinase inhibitor, targets multiple receptors, including the vascular endothelial growth factor receptor, platelet-derived growth factor receptor, various Raf kinases, to exert its anti-tumor effects (Li et al., 2020; ECoLCoCSoC, 2022). Previous studies have identified adverse events associated with Donafenib, including hand-foot skin reactions, diarrhea, elevated aspartate aminotransferase levels, and decreased white blood cells counts (Qin et al., 2021). However, reports on adverse ocular manifestations of Donafenib are not yet comprehensive.

Vogt-Koyanagi-Harada (VKH), characterized as bilateral granulomatous panuveitis, is an inflammatory condition affecting both eyes and can lead to symptoms such as meningeal irritation, hearing impairment, vitiligo, and hair loss or whitening (Greco et al., 2013; Lavezzo et al., 2016). The precise causes and mechanisms of VKH remain unclear. However, studies have indicated that the overexpression of HLA-DR4/DR53, along with certain environmental factors such as cytomegalovirus infections, could activate the adaptive immune response, leading to the activation of Th1 and Th17 cells (Greco et al., 2013; Du et al., 2016; O'Keefe and Rao, 2017). Ultimately, these immune responses culminate in an acute autoimmune reaction affecting multiple organs, primarily due to the direct targeting of self-antigens, such as tyrosinase, which are expressed by melanocytes (Du et al., 2016; O'Keefe and Rao, 2017; Sakata et al., 2014).

We report a case of VKH-like uveitis caused by Donafenib in the treatment of primary hepatocellular carcinoma, which has not been reported before, emphasizing the need for ophthalmological follow-ups for tumor patients receiving Donafenib and other novel targeted inhibitors.

## Case presentation

A 55-year-old man presented with a complaint of persistently reduced vision for over a month. The patient was diagnosed with hepatocellular carcinoma (stage Ia, China Liver Cancer Staging System (Xie et al., 2020)) in right lobe, and underwent laparoscopic hepatectomy and cystectomy 3 years ago. Following surgery, he was treated by Entecavir Dispersible Tablets (RuiFuEn, 0.5 mg/tablet, Suzhou Dawnrays Pharmaceutical Co., Ltd.) orally 0.5 mg/d. However, the disease relapsed one and a half years later, manifesting as indistinct shadows on liver in the CT examination. Consequently, he underwent radiofrequency ablation, leading to his current treatment with a combination of Donafenib (Zepsun, 100 mg, Suzhou Zelgen Biopharmaceuticals Co., Ltd) and Sintilimab (PD-1 inhibitor, 100 mg, Innovent) instead of Entecavir Dispersible Tablets. He was administered Donafenib orally 400 g/d, and the Sintilimab was administered by intravenous injection at a dose of 200 mg every 21 days. Unfortunately, after 18 months of using Donafenib and the Sintilimab, he experienced sporadic white patches on his body, tinnitus, headaches, and mild bilateral vision reduction. He sought medical attention because his vision had dramatically worsened in the past 4 weeks. The patient was diagnosed as hepatitis B for over 10 years, and had no history of systematic disease like hypertension, diabetes, drug allergy.

**FIGURE 1:**
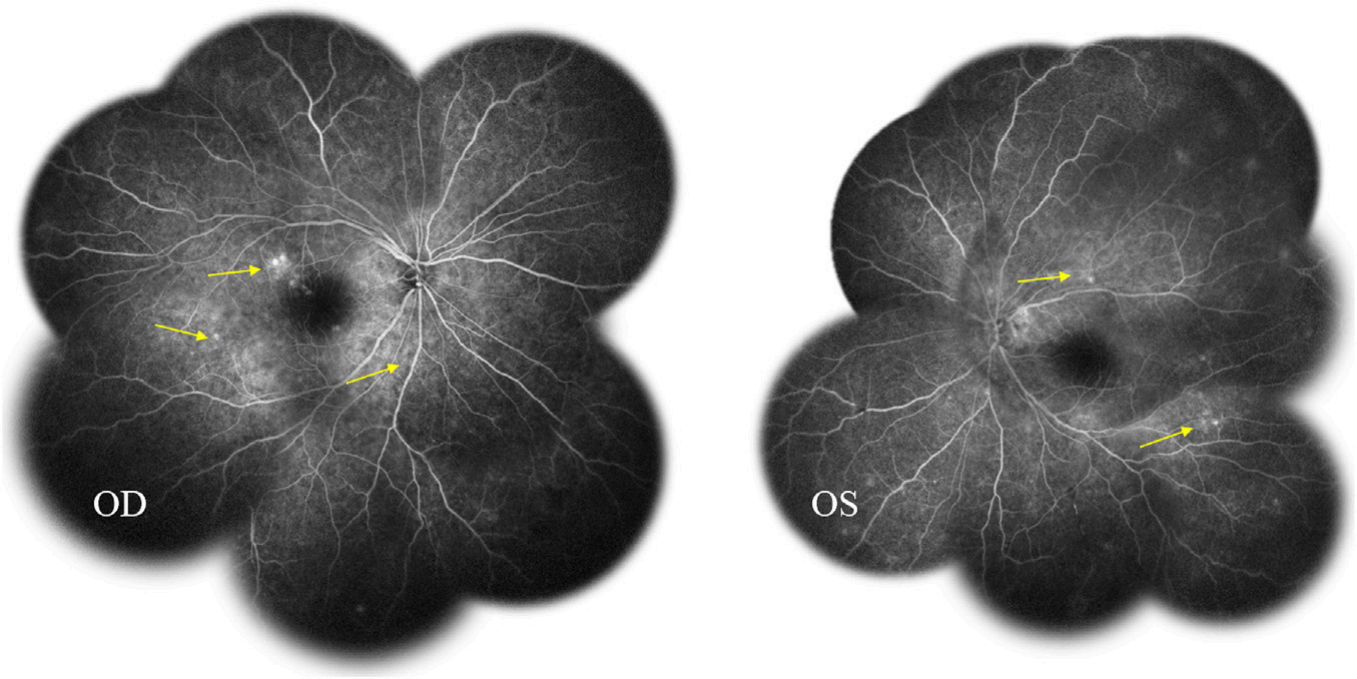
Fundus fluorescein angiography (FFA) displayed multifocal hyperfluorescent lesions at the level of retinal pigment epithelium pooled into the sub-neurosensory retinal space (*arrows*).

**FIGURE 2:**
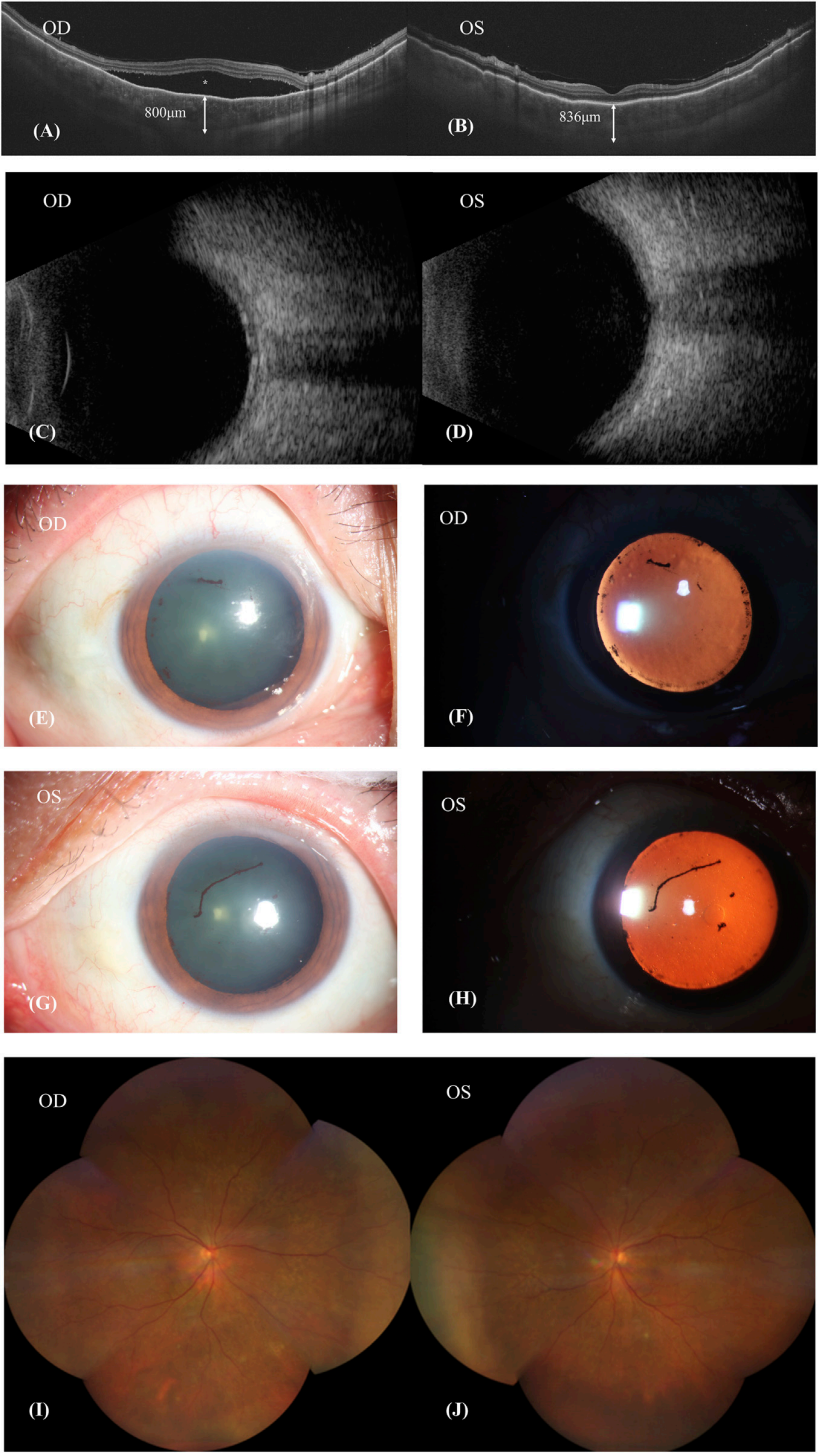
Patient’s first visit. **(A,B)**: SS-OCT showed the neurosensory retinal detachment (NSD) in the right eye (*asterisk*), choroid folds and thickened choroid in both eyes. The subfoveal choroid thickness (SFCT) were 800 μm in the right eye and 836 μm in the left eye. **(C,D)**: Ultrasound B scan showed thickening of choroid and clear eyeballs. **(E–H)**: Both eyes showed signs of iris synechiae and anterior uveitis with fibrin formation. **(I,J)**: Patient’s fundus image was basically normal.

Fundus fluorescein angiography (FFA) and swept-source optical coherence tomography (SS-OCT, SVison) revealed VKH-like manifestations, including exudative retinal detachment and choroidal thickening. The FFA images demonstrated multiple focal areas of pinpoint hyperfluorescence, along with pooling indicative of neurosensory detachment (NSD) and disc leakage in both eyes (Figure 1). The SS-OCT images of the patient revealed NSD in the right eye (Figure 2A), as well as choroidal folds and thickened choroid in both eyes (Figures 2A, B). We measured the patient’s subfoveal choroid thickness (SFCT), which was 800 μm in the right eye and 836 μm in the left eye.

The patient’s BCVA is 20/200 in his right eye and 20/80 in his left eye. Ultrasound B-scan imaging revealed a clear vitreous body but demonstrated choroidal thickening in both eyes (Figures 2C, D). Ophthalmic anterior segment imaging displayed signs of iris synechiae and anterior uveitis, along with fibrin formation (Figures 2E–H). However, the patient’s fundus imaging appeared basically normal (Figures 2I, J). Following comprehensive examinations, it was considered that the patient had uveitis and was positive for the HLA-B27 antigen. The patient’s erythrocyte sedimentation rate (ESR) and C-reactive protein (CRP) levels were elevated at 42.0 mm/h and 7.01 mg/L, respectively, indicative of inflammation. Evidence of an active immune response was indicated by elevated levels of immunoglobulins IgG (19.00 g/L), IgA (5710 mg/L), and IgE (612 IU/mL). Additionally, he tested positive for hepatitis B surface antigen (HBsAg) and hepatitis B e antigen (HBeAg). Analysis of his intraocular fluid revealed high levels of interleukins IL-6 (6090.44 pg/mL), IL-8 (1357.48 pg/mL), and IL-10 (36.83 pg/mL), indicating ocular inflammation. Considering the necessity of Sintilimab for hepatocellular carcinoma and previous reports on the ocular side effectives from sorafenib whose mechanism was similar to Donafenib, we recommended discontinuation of Donafenib and regular follow-ups at the eye clinic.

**FIGURE 3:**
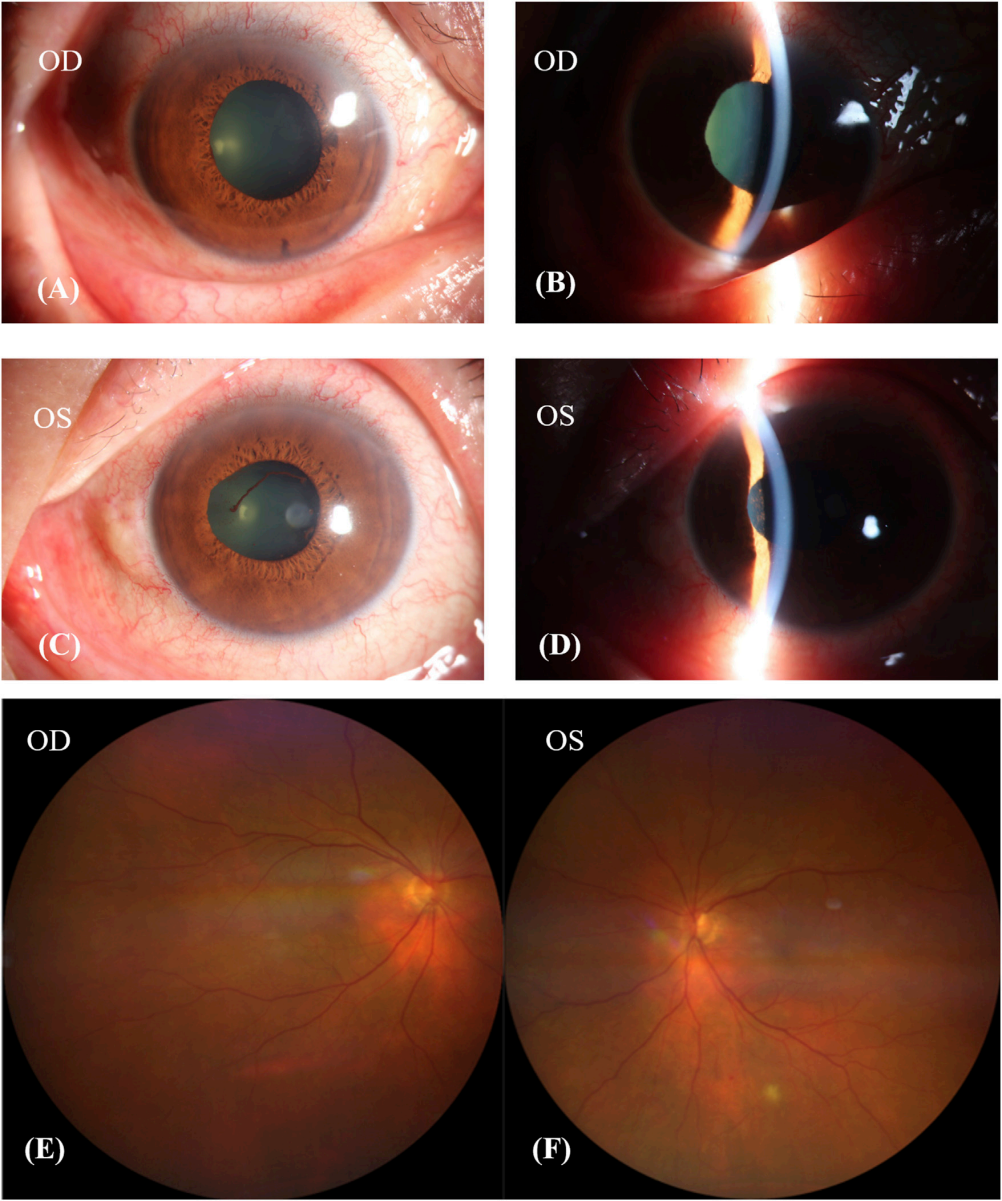
Patient’s condition after discontinuing donafenib 1 week. **(A–D)**: The pupil and iris had synechiae in both eyes, and the anterior uveitis of left eye. **(E,F)**: Patient’s fundus image showed choroidal depigmentation, sun-set fundus.

One week after discontinuing Donafenib, his BCVA remained unchanged, but he experienced relief from his headache and tinnitus. Examination revealed synechiae involving the pupil and iris in both eyes, and anterior uveitis was observed in the left eye (Figures 3A–D). His fundus image showed choroidal depigmentation as sun-set fundus (Figures 3E, F). Considering his VKH-like symptoms, we initiated treatment with oral glucocorticoids. This involved weekly tapering by 5 mg of prednisolone acetate orally, starting at a dose of 80 mg (1 mg/kg body weight). Additionally, neocyspin (25 mg) was prescribed to be taken orally twice a day (morning and evening) for 3 months.

**FIGURE 4:**
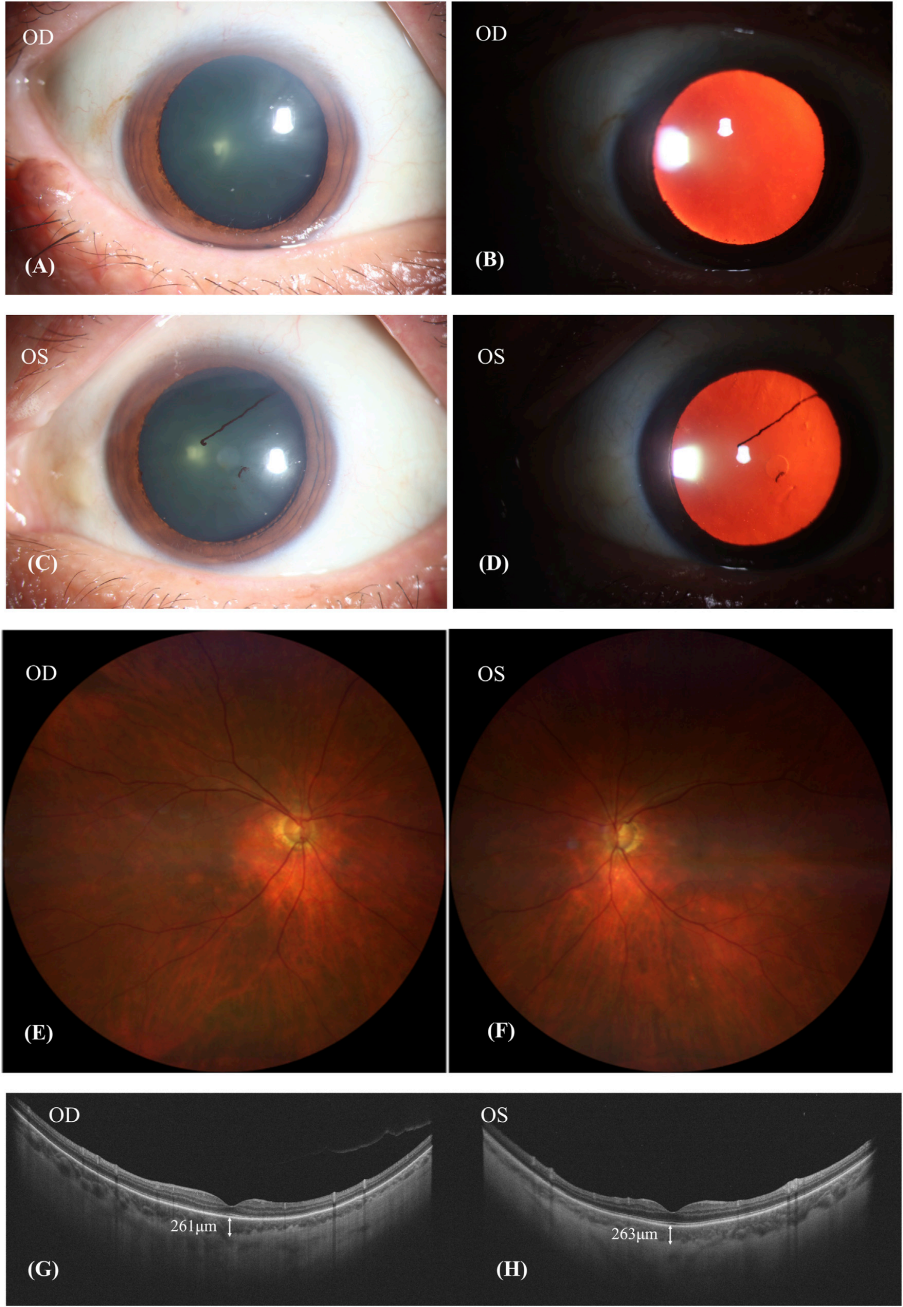
Patient’s condition after discontinuing donafenib and receiving oral glucocorticoid and neocyspin for 3 months. **(A–D)**: The obvious red-light reflex suggested that the fundus of the patient had a sunset-glow change, and the lens surface of the left eye had anterior synechiae. **(E,F)**: A pale nerve surrounded by red-orange choroidal depigmentation was referred to as a “sunset-glow” fundus. **(G,H)**: SS-OCT showed retina flatted. The choroid was thinned out and the choroidoscleral border was visible. The subfoveal choroid thickness (SFCT) was 261 μm in the right eye and 263 μm in the left eye.

After discontinuing Donafenib and receiving oral glucocorticoid and neocyspin for 3 months, the patient’s BCVA improved to 20/30 in the right eye and 20/40 in the left eye. Ophthalmic anterior segment imaging showed the obvious red-light reflex, suggesting fundus of the patient had a sunset-glow change, and the lens’ surface of the left eye had anterior synechiae (Figures 4A–D). The fundus imaging depicted a pale optic nerve surrounded by red-orange choroidal depigmentation, characteristic of a “sunset-glow” fundus appearance (Figures 4E, F). He responded well to the oral corticosteroid therapy, as evidenced by the resolution of exudative detachment and diminishing fluid observed in the SS-OCT (Figures 4G, H). The SS-OCT images showed a flattened retina and thinning of the choroid, where the choroidoscleral border became visible. The SFCT was 261 μm in the right eye and 263 μm in the left eye.

With the improvement of the patient’s ocular symptoms, we collaborated with the multidisciplinary team to continue the treatment of hepatocellular carcinoma with the lowest effective oral dose of Donafenib (200 mg/day) and recommended regular ophthalmological follow-ups. One month after resuming Donafenib, the patient returned for ophthalmologic examinations, which indicated that his condition remained stable. His BCVA was 20/25 in the right eye and 20/30 in the left eye.

## Discussion and conclusion

In the literature, we firstly reported case of VKH-like uveitis caused by Donafenib. Various types of drugs result in DIU, including cidofovir (Davis et al., 1997; Lopez et al., 2006), rifabutin (Bhagat et al., 2001; Trone et al., 2018), bisphosphonates (Etminan et al., 2012; Chartrand et al., 2023), sulfonamides (Arola et al., 1998; Pathak and Power, 2007), nonsteroidal anti-inflammatory drugs (Mandeville et al., 2001; Okafor et al., 2017), tumor necrotic factor-α inhibitors (Ramos-Casals et al., 2008; Seve et al., 2012), fluoroquinolones (Forooghian et al., 2013; Sandhu et al., 2016), immune checkpoint inhibitors (Arai et al., 2017; Conrady et al., 2018), and BRAF/MERK inhibitors (Moorthy et al., 2018; Agarwal et al., 2020; Dimitriou et al., 2021). Additionally, the advent of targeted therapy has given rise to cases of targeted drug-induced uveitis. Uveitis has been reported as a consequence of using sorafenib and sunitinib (Fraunfelder and Fraunfelder, 2018). In the treatment of metastatic melanoma, the BRAF inhibitor vemurafenib has been associated with VKH-like symptoms (Lim et al., 2018; Xiaotong et al., 2022), resulting in drug-induced uveitis and secondary glaucoma (Xiaoli et al., 2022).

Given the patient’s medical history and clinical presentations, we considered the possibility of DIU. The patient received PD-1 immune therapy, known to cause VKH-like responses (Cunningham et al., 2020), concurrently with Donafenib. It was crucial to determine which drug was responsible for the VKH-like manifestations in this case or the potential interaction of the two drugs. Considering the potential risks associated with novel targeted therapies, and Sorafenib with similar mechanism to Donafenib, had potential risk of inducing uveitis reported before (Fraunfelder and Fraunfelder, 2018), we opted to discontinue Donafenib for 1 week to evaluate whether this would result in an improvement in the patient’s fundus condition. After discontinuing Donafenib, the patient reported improved headache and tinnitus. We initiated corticosteroid therapy to confirm the diagnosis. Three months after discontinuing Donafenib, we observed an improvement in the VKH-like manifestations. As a result, we speculated that the VKH-like symptoms were triggered by the use of Donafenib.

Donafenib is employed as a first-line targeted therapy for hepatocellular carcinoma in China (LCCoCMD, 2022). It is an oral small molecule inhibitor, effectively suppressing tumor growth and angiogenesis, achieved by targeting several receptor tyrosine kinases, including those linked to vascular endothelial growth factor and platelet-derived growth factor. Additionally, Donafenib targets various Raf kinases and the Raf/MEK/ERK signaling pathway, leading to dual inhibition and multi-target blockade, thereby effectively combating cancer (Li et al., 2020; Qin et al., 2021; ECoLCoCSoC, 2022). During the follow-up, except for ophthalmological performance, we discovered that the patient experienced tinnitus, headache, hearing loss, and sporadic white patches, which are typical symptoms of VKH. After stopping the use of Donafenib and starting corticosteroid therapy, his symptoms showed a positive response to it. Consequently, we speculated that the use of Donafenib led to the development of uveitis, which mimicked the VKH effects on ophthalmological performance.

Although the exact mechanism remains unclear, we have developed several hypotheses to explain this process. One hypothesis posits that Donafenib’s inhibition of the BRAF pathway may activate the immune system, potentially triggering an immune reaction that results in VKH-like uveitis (Xiaotong et al., 2022). Furthermore, the anti-tumor effect of BRAF inhibitors leads to widespread apoptosis of tumor cells, resulting in the release of a significant number of antigens (Apivatthakakul et al., 2020). This may cause systemic hypersensitivity and ocular involvement, manifesting as uveitis.

Our study had several limitations. Firstly, we discontinued Donafenib for only 1 week, which was not sufficient to fully observe the progression of the patient’s condition. Secondly, our study included only one case and future research should involve larger sample sizes. Thirdly, it was challenging to discern the potential interaction between Donafenib and Sintilimab by stopping Donafenib only. However, in the real clinical practice, Sintilimab was essential for the patient’s therapy and we could not discontinue or alter it. In the future research, we aim to explore the potential interaction Donafenib and Sintilimab using cell lines and animal models.

In conclusion, we reported the first case of VKH-like uveitis induced by Donafenib, underscoring the necessity of ophthalmological follow-ups during targeted therapy. The patient’s condition improved following the discontinuation of Donafenib, and the addition of prednisolone acetate further accelerated this improvement. We successfully observed the entire progression of fundus changes associated with the VKH-like uveitis. To definitively determine the mechanism by which Donafenib causes VKH-like uveitis, further rigorous experiments and more substantial evidence are required.

## Data Availability

The raw data supporting the conclusions of this article will be made available by the authors, without undue reservation.
